# The Role of the Duffy Blood Group Antigens in Renal Transplantation and Rejection. A Mini Review

**DOI:** 10.3389/ti.2023.11725

**Published:** 2023-10-13

**Authors:** Dana Hariri, Jozsef Bordas, Matthew Elkins, Brian Gallay, Zhanna Spektor, Reut Hod-Dvorai

**Affiliations:** ^1^ Department of Pathology, SUNY Upstate Medical University, Syracuse, NY, United States; ^2^ Department of Medicine, SUNY Upstate Medical University, Syracuse, NY, United States

**Keywords:** Duffy, red blood cells (RBC), renal transplant, rejection, alloantibody

## Abstract

Finding a compatible donor for kidney transplant candidates requires overcoming immunological barriers such as human leukocyte antigens (HLA) compatibility and ABO compatibility. Emerging data suggest a role for red blood cell antigens (RCA) in renal transplant outcomes. The incidence of RCA alloimmunization is high in chronically transfused individuals, such as end stage renal disease patients, but whether antibodies to RCA can mediate renal graft rejection remains debatable. The Duffy blood group antigens (Fy) has been shown to be expressed in the kidney, among other tissues. There are some data to suggest that donor-recipient Fy mismatches may increase the risk for chronic allograft damage and that anti-Fy antibodies may be involved in renal graft rejection, however, while it is routine to screen renal transplant candidates for ABO antigens, detailed RCA phenotyping of the donor kidney is not routinely tested. In this paper, we review the current data on the role of Fy in renal transplantation and discuss the potential mechanisms of its biological function.

## Introduction

Finding a compatible donor for kidney transplant candidates requires overcoming immunological barriers such as human leukocyte antigens (HLA) compatibility and ABO compatibility, which may prolong time to transplant. Emerging data suggest a role for red blood cell antigens (RCA) in renal transplant outcomes [1–3]. As of July 2023, there are 45 recognized blood group systems, many of which are expressed on tissue other than the surface of red blood cells (RBC) [4]. The functions of these antigens are not all fully understood but some function as chemokine receptors and others as membrane transporters [5]. Of the 45 recognized blood groups the kidney is known to express Duffy, Lewis, Kidd and MNS blood groups [1, 5]. Antibodies to RCA (other than ABO) are usually not naturally occurring and require exposure for development. Transfusions are the main source of exposure for most RCA with each transfusion event increasing the likelihood of alloimmunization by 0.2%. The probability of developing RCA allosensitization is dependent on multiple factors including underlying disease, immunogenicity of the antigen, dose and frequency of transfusion, age, and gender among other factors [6]. The incidence of RCA alloimmunization in multiply transfused individuals, such as end stage renal disease patients requiring frequent transfusions, can be as high as 60% [2, 6].

Whether antibodies to RCA can mediate renal graft rejections remains debatable. Various studies produced mixed results when looking at retrospective data and trying to correlate presence of antibodies with kidney graft survival [1, 3]. There are some data to suggest that donor-recipient Duffy blood group (Fy) mismatches may increase the risk for chronic allograft damage [3]. However, while it is routine to screen renal transplant candidates for ABO antigens, detailed RCA phenotyping of the donor kidney is not routinely tested. In this paper, we review the current data on the role of Fy in renal transplantation and discuss the potential mechanisms of its biological function.

## The Duffy Blood Group Antigens

**FIGURE 1:**
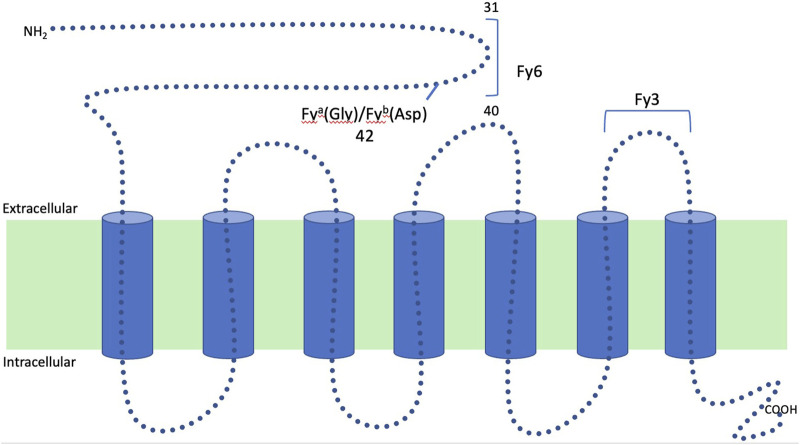
Structure of the Duffy glycoprotein: Duffy is a seven transmembrane glycoprotein that has multiple epitopes for which antibodies can be formed. It is encoded on chromosome 1 by two codominant alleles (FY*A and FY*B). These two alleles differ by a single nucleotide polymorphism at position 125 (G/A) resulting in the presence either of glycine or aspartic acid in position 42 of polypeptide chain that gives rise to the two antigens, Fy^a^ and Fy^b^.

The Duffy blood group system was first described in 1950 in a patient named Duffy who presented with hemolytic transfusion reactions after having received multiple transfusions [7, 8]. Fy is a seven transmembrane domain glycoprotein that has multiple epitopes for which antibodies can be formed. It is encoded on chromosome 1 by two codominant alleles (FY*A and FY*B). These two alleles differ by a single nucleotide polymorphism at position 125 (G/A) resulting in the presence either of glycine or aspartic acid in position 42 of the polypeptide chain that gives rise to the two antigens, Fy^a^ and Fy^b^ (Figure 1). Depending on the alleles present, four main phenotypes may be found in the population: Fy(a+ b−), Fy(a− b+), Fy(a+ b+) and Fy(a− b−), although the phenotypic expression in tissue versus blood can vary depending on the specific genotype (Table 1) [7, 9]. The most common antigens are Fy^a^ and Fy^b^, but Fy3, Fy5, and Fy6 have also been described [5]. Antibodies for Duffy are almost never naturally occurring and are a result of exposure to the antigen. Antibodies to Fy^a^ are more frequently seen than antibodies to Fy^b^ by about a 20-fold increase. These antibodies are predominately IgG1 with a small percentage of presentations consisting of IgM (25%) and IgG2 (18%) [5, 7]. Antibodies against both Fy^a^ and Fy^b^ cause immediate and delayed hemolytic transfusion reactions and have been associated with hemolytic disease of the fetus and newborn [5].

**TABLE 1:** Examples of Duffy genotype and phenotypic expression in blood vs. tissue.

Genotype	Phenotype (blood)	Phenotype (tissue)
FY*A/FY*A	Fy(a+, b−)	Fy(a+, b−)
FY*B/FY*B	Fy(a−, b+)	Fy(a−, b+)
FY*A/FY*B	Fy(a+, b+)	Fy(a+, b+)
FY*02N.01/FY*02N.01 ^a^	Fy(a−, b−)	Fy(a−, b+)
FY*02N.02/FY*02N.02 ^b^	Fy(a−, b−)	Fy(a−, b−)

^a^ This genotype represents the GATA box mutation (erythrocyte silent phenotype).

^b^ This genotype is one on many that cause the true null phenotype which prevents expression of Duffy on both blood and tissue. For more information, please refer to Höher et al. [9].

### Duffy Expression in Different Ethnic Populations

Duffy expression varies greatly in different ethnicities. Caucasians and Asians have near 100% expression on their RBC, whereas only 33% of African Americans (AA) express Fy on their RBC [5]. Variants of the Duffy blood group system, their phenotype and genotype frequencies are reviewed elsewhere [9]. An explanation to this difference in expression can be provided by Duffy’s functional role as a receptor for the malaria parasite *Plasmodium vivax*. RBC lacking Fy are more resistant to invasion from this parasite. The lack of expression of Fy in AA is caused by a point mutation in the GATA box promoter of the Fy^b^ allele (FY*02N.01; 1–67 T>C), which prevents expression of Duffy protein only on RBC [5, 7]. This mutation is seen with a high incidence of up to 95% in western and southwestern sub-Saharan Africa and correlates with a low prevalence of Plasmodium vivax [10]. However, individuals carrying this mutation rarely develop Fy3 alloantibodies since Fy expression is preserved on other tissues. The true Duffy null phenotype, resulting in complete loss of Fy expression on all tissues, is seen in patients homozygous for either a point mutation or deletion in exon 2 (e.g., FY*02N.02) which introduces a stop codon for the Duffy gene in all tissues (see example in Table 1). This phenotype allows for production of a Fy3 alloantibodies. Fy3 antibodies where first described in 1971 and have since been recognized with more case reports being presented [11–13]. The prevalence of Duffy null phenotype is unknown due to its rarity [5].

### Duffy Antigen Receptor for Chemokines (DARC)

The Duffy antigen was identified as a transmembrane glycoprotein coupled receptor and was renamed the Duffy antigen receptor for chemokines (DARC) [14, 15]. DARC is found on the surface of RBC as well as the endothelium of postcapillary venules of lymph nodes, spleen, the kidney, and other organs [14]. DARC is shown to bind many different chemokines including Interleukin 8 (IL-8), monocyte chemoattractant protein 1 (MCP-1), CXC chemokines and regulated on activation normal T-cell expressed and secreted (RANTES) [16, 17]. Since no downstream effects have been identified after chemokine binding to DARC, it was hypothesized that it acts as a chemokine sink by attracting and binding chemokines, therefore reducing their bioavailability [17]. When challenged with lipopolysaccharide induced endotoxemia, DARC knockout mice showed an increase in chemokine production within multiple organs compared to control mice, supporting DARC’s role in regulating the inflammatory response [18]. DARC has been shown to play a role in modulating immune responses in multiple diseases such as HIV, COVID-19 and atherosclerosis; given that, it is reasonable to think that DARC plays a role in renal transplantation [7].

### DARC’s Role in Renal Transplantation

One way in which DARC can affect renal transplant outcomes is by acting as a minor histocompatibility antigen. A Lerut et al. did a retrospective study of 370 renal transplant recipients comparing Fy matched (*n* = 239) versus mismatched (*n* = 131) donor-recipient pairs and correlated Fy mismatch status with histologic findings of kidney biopsies and overall survival [3]. Although graft survival and acute histologic lesions were similar between the groups, the study found increased incidence of chronic histologic lesions (e.g., interstitial fibrosis, tubular atrophy and intimal fibrosis) in Fy mismatched recipients despite the fact that the matched recipients had longer cold ischemia times and higher sensitization status (as indicated by PRA). Both FY*A mismatches and FY*B mismatches were associated with chronic lesions. However, the FY*B mismatched group showed only increased intimal fibrosis which may have been attributed to older age in this group compared with the Fy matched group. The FY*A mismatched group had no age or other demographic differences compared with the Duffy matched group, yet this group showed significantly more chronic histologic changes. The difference between FY*A and FY*B mismatches was attributed to the higher immunogenicity of FY*A compared to FY*B [3]. Altogether, these results support Duffy’s role as a minor histocompatibility antigen.

Another way in which DARC can affect renal transplant outcomes is through its expression levels. DARC expression is upregulated in the kidney in response to multiple environments of cell injury including HIV, nephropathy, hemolytic uremic syndrome, delayed graft function (DGF), ischemia reperfusion injury (IRI), and acute renal allograft rejection [16]. A study by Segerer et al. found upregulation of DARC positive venules in the setting of acute rejection post-transplant compared to pre-transplant biopsies [14]. This increase was even more prominent in cases with signs of both cellular and humoral rejection [14]. Akalin et al. studied a retrospective cohort of 117 kidney transplant recipients who were categorized based on their RBC Fy phenotype. The study showed a strong association between Fy(a−, b−) patients with DGF and graft failure, indicating that DARC may decrease the inflammatory response during DGF, causing DARC-negative patients to be more susceptible to DGF [16].

## Discussion

**FIGURE 2:**
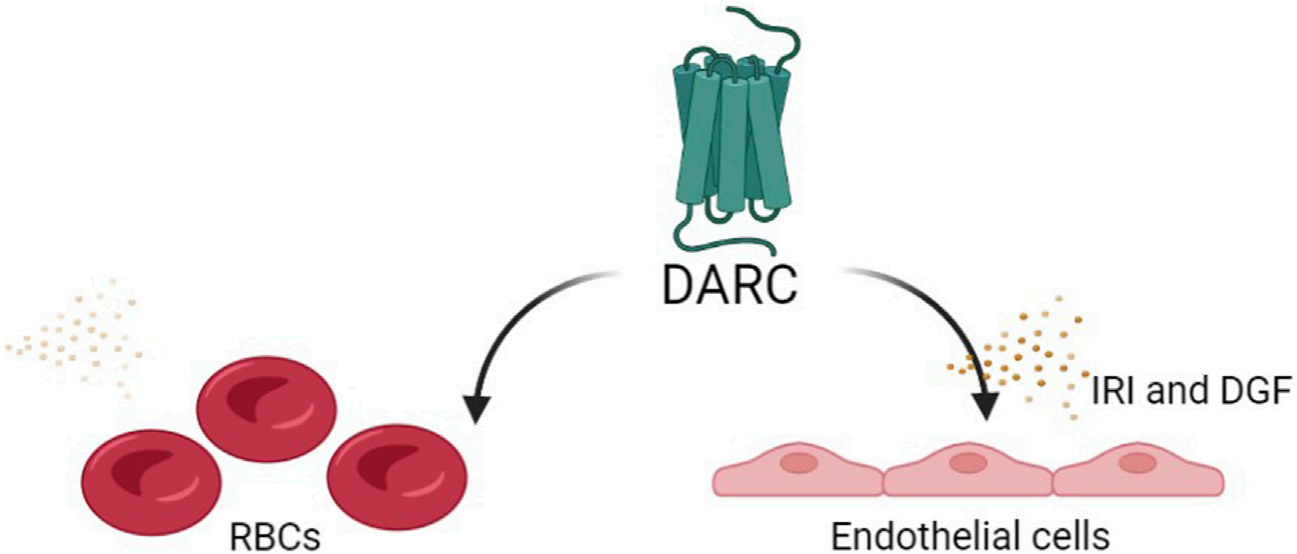
The potential functional role of DARC in recruitment of inflammatory cells: The functional role of DARC in recruitment of inflammatory cells is still not fully understood, and several mechanisms have been suggested: 1) DARC helps presenting chemokines on the endothelial cells to leukocytes expressing corresponding receptors; 2) The binding of chemokines to DARC helps creating a gradient flow to keep an influx of chemokines coming to the site; 3) Neutralization of bound chemokines [19, 20]. The question remains whether this upregulation is an attempt to bind and neutralize chemokines and control the inflammation or is this an attempt to recruit more inflammatory cells? It is also possible that DARC is able to pursue different functions, proinflammatory in some scenarios and anti-inflammatory in others, according to the spatial and the temporal context.

The functional role of DARC in recruitment of inflammatory cells is still not fully understood, and several mechanisms have been suggested: 1) DARC helps presenting chemokines on the endothelial cells to leukocytes expressing corresponding receptors; 2) The binding of chemokines to DARC helps to create a gradient flow to keep an influx of chemokines coming to the site; 3) Neutralization of bound chemokines [19, 20]. Although the mechanism of DARC is not clear, there is evidence of upregulation of DARC on peritubular capillaries during both humoral and acute cellular rejection episodes [19]. The question remains whether this upregulation is an attempt to bind and neutralize chemokines and control the inflammation or is this an attempt to recruit more inflammatory cells? It is important to note that most of the studies investigating Duffy’s role in renal transplantation rely on RBC Fy phenotyping, which may be different from the Fy phenotype in the renal tissue. It is possible that DARC is able to pursue different functions according to the spatial and the temporal context (Figure 2).

The idea that anti-Fy antibodies may participate in renal allograft rejection is supported by a case report by Watorek et al. The authors reported a 41 years-old Caucasian woman, Fy(a− b+) phenotype, who had anti-Fy^a^ antibodies detected 2 years prior to her transplant, although at time of transplant her anti-Fy^a^ antibodies were undetectable. Her post-transplant hospital course was complicated by DGF and a biopsy at 26 days post-transplant revealed acute rejection, both of which are associated with upregulation of DARC expression in the kidney [14, 19]. Two months post-transplant her serum tested positive for anti-Fy^a^ antibodies and a repeat biopsy at 3 months showed acute and C4d+ antibody-mediated rejection in the absence of HLA donor-specific antibodies (DSA). The authors concluded that the unfavorable outcome of her transplant is a result of the presence of antibodies to Fy^a^ [21]. At the time of writing this paper, this is the only case report found linking the cause of kidney rejection to anti-Fy antibodies. However, case reports have suggested the involvement of antibodies to other RBC groups in renal allograft rejection [22–25].

At our center, we recently evaluated a 30 years-old African female with a history of multiple RBC transfusions due to anemia for a kidney transplant. Her HLA panel reactive antibody (PRA) was 0%. An ABO type and screen identified antibodies to Fy3, JkB, E, C, and K. The finding of the anti-Fy3 suggested that this patient carries the very rare true Fy null phenotype. Despite her ability (albeit limited) to receive transfusions due to the absence of Fy on RBC in most AA, her ability to find a compatible kidney donor is virtually impossible due to the presence of Fy on nearly every donor kidney. Even with a perfectly HLA and ABO matched donor, there is a potential for rejection due to anti-Fy3 reactivity and therefore this patient was deemed not suitable for transplantation at our center. Currently, there are no guidelines for donor-recipient RCA matching, mostly since more data is needed and since this will narrow down the donor pool for many transplant candidates. However, a more detailed screening process for RBC antibodies and RCA phenotyping of the donor may be warranted in patients experiencing antibody-mediated rejection (ABMR) with no HLA-DSA, especially if a recent post-transfusion reaction was observed prior to the diagnosis of ABMR. RCA antibody characterization might also be helpful prior to re-transplant following an ABMR involving RCA antibodies, and in cases where the recipient has a rare null phenotype. Differentiating between the true null and the erythrocyte silent mutations can be achieved by utilizing DNA-based typing instead of serologic phenotyping.

In conclusion, most of the emphasis in overcoming immunological barriers in solid organ transplantation is rightfully put on matching donors and recipients for HLA and ABO, however, the Duffy blood group system can present a unique and rare barrier to transplantation and potentially impact transplant outcomes.

## Abbreviations

AA, African American; ABMR, antibody-mediated rejection; DARC, Duffy antigen receptor for chemokines; DGF, delayed graft function; Fy, Duffy blood group antigen; HLA, human leukocyte antigens; IRI, ischemia reperfusion injury; RCA, red blood cell antigen; RBC, red blood cells.
